# Temperature responsive aluminum manganese doped carbon dot sensors for enhanced electrical conductivity with DFT calculations

**DOI:** 10.1038/s41598-025-04531-1

**Published:** 2025-06-05

**Authors:** Mohamed El-Nasharty, Mohamed El-Sakhawy, Hebat-Allah S. Tohamy

**Affiliations:** 1 Microwave Physics and Dielectrics Department, Physics Research Institute, National Research Centre, 33 El Bohouth St, Dokki, 12622 Giza Egypt; 2 Cellulose and Paper Department, National Research Centre, Dokki, 12622 Giza Egypt

**Keywords:** Electrical conductivity, Aluminum/manganese-doped carbon quantum dots (Al-Mn/CQDs), Sugarcane bagasse, Carboxymethyl cellulose (CMC), Microwave-assisted synthesis, Agricultural waste valorization, Environmental sciences, Chemistry, Energy science and technology, Materials science, Nanoscience and technology

## Abstract

Agricultural wastes provide abundant cellulosic by-products, making them excellent candidates for sustainable material production. In this study, sugarcane bagasse was used to synthesize aluminum/manganese-doped carbon quantum dots (Al-Mn/CQDs) through a microwave-assisted process. Aluminum doping and subsequent thermal treatment progressively reduced the band gap of manganese-doped carbon quantum dots from 1.21 eV to 0.7 eV and 0.3 eV, respectively, demonstrating a tunable electronic structure with implications for applications requiring specific emission wavelengths. The resulting CQDs exhibit a spherical morphology (1.95–2.05 nm) and, upon aluminum incorporation, form uniform sheet-like structures decorated with these particles. Optical analysis shows a notable improvement in fluorescence quantum yield, reaching up to 42.65% at elevated synthesis temperatures, and a narrow full width at half maximum, demonstrating strong potential for bioimaging and sensing applications. Aluminum incorporation into Mn/CQDs lowers the LUMO energy level from − 0.12459 to − 0.14838 eV, indicating that aluminum creates or modifies acceptor states to favor electron acceptance. Moreover, the total energy decreases from − 1638.16 au in Mn/CQDs to − 1874.34 au in Al-Mn/CQDs, underscoring the enhanced stability and favorable formation of the aluminum-modified carbon dots. Density functional theory (DFT) calculations reveal a lower energy gap (0.0482 eV), higher softness (20.74 eV), and enhanced charge transfer, findings confirmed by stable and low-impedance conductivity across a wide frequency range. These properties make Al-Mn/CQDs ideal for antistatic protection, electromagnetic interference shielding, and RLC bridge calibration, while their temperature-sensitive behavior also shows promise for temperature sensing applications.

## Introduction

Fossil fuels have dominated energy production, accounting for 74%, while renewables have contributed 26%^1,2^. Renewable energy is poised to grow due to its lower carbon footprint, which helps address environmental concerns^3,4^. However, storage solutions are crucial for grid stability, especially with intermittent renewables. Various systems, ranging from large-scale pumped hydro to small-scale batteries offer potential solutions^5,6^. The global energy sector is undergoing a profound transformation, driven by the urgent need to address climate change and ensure a sustainable energy supply^7–9^. Therefore, the development of efficient biomass-derived energy storage technologies is crucial for the successful transition to a sustainable energy future^10^.

Carbon quantum dots (CQDs) are tiny, spherical carbon particles with unique properties^11,12^. They have a core of carbon atoms and a surface covered with functional groups like carboxylic acids, alcohols, and aldehydes. Unlike other carbon materials, CQDs are extremely small and h exhibit superior electron transfer, light-emitting properties, and quantum effects^13–15^. They’re also safer for the environment and easier to work with than traditional semiconductor quantum dots. CQDs can be made through various methods, including top-down and bottom-up approaches. Top-down methods break down larger carbon structures into nanoscale dots using techniques such as laser ablation, arc-discharge, chemical oxidation, and ultrasonic exfoliation^16^. In contrast, bottom-up techniques build CQDs from molecular precursors through hydrothermal or solvothermal synthesis, microwave-assisted synthesis, combustion methods, and pyrolysis^17–20^. To further enhance the electrical properties of CQDs, particularly for applications in sensing and electronic devices, doping with metal ions has emerged as a promising strategy^21,22^. Metal ion doping of CQDs can substantially modify their optical, electronic, and magnetic properties by altering both the electron density distribution and the energy gap^23^. The one-pot hydrothermal carbonization method synthesized nitrogen and aluminum co-doped carbon dots (N/Al-CQDs) with a photoluminescence quantum yield of 28.7%. These N/Al-CQDs were further developed as a fluorescence sensor for the highly selective and sensitive detection of Mn(VII) ions^24^. In addition, co-doping nitrogen and nitrogen-sulfur carbon dots with first-row transition metal ions (Mn²⁺, Fe²⁺, Co²⁺, Ni²⁺, Cu²⁺, and Zn²⁺) enhanced their capacitance values, which ranged from 0.5 µF/cm² to 2.6 µF/cm², consistent with those reported for other carbon-based materials such as graphene^25^.

Manganese oxide decorated carbon quantum dots (CQDs) derived from agricultural waste, demonstrating exceptional supercapacitor performance, were successfully synthesized. This innovative approach yielded a hybrid material with a high specific capacitance of 612 F g⁻¹ at 1 A g⁻¹ and 657.1 F g⁻¹ at 5 mV/s, showcasing the synergistic benefits of integrating Mn with CQDs. Furthermore, the synthesized electrodes exhibited remarkable cycling stability, retaining 96.3% of their initial capacitance after 5000 cycles with a coulombic efficiency of 98%. These findings highlight the potential of utilizing agricultural waste for sustainable energy storage, offering a path towards a circular economy^26^. Similarly, the synthesis of manganese-doped carbon dots (Mn/CD) also demonstrated enhanced electrochemical properties. The incorporation of manganese ions into the CD matrix led to improved electrical conductivity and charge storage capabilities, indicating the effectiveness of metal doping in tailoring CD properties for energy storage applications^27^. These works, underscore the promising avenue of metal-modified carbon-based materials for advancing high-performance supercapacitors^28^.

We focus, for the first time, on the synergistic incorporation of manganese (Mn) and aluminum (Al) ions into the CD matrix. We hypothesize that the introduction of these dopants will induce structural modifications and electronic alterations, leading to a significant improvement in electrical conductivity. Notably, instead of the conventional co-precipitation method, we employ a microwave-assisted synthesis approach. This method offers several advantages, including rapid heating, uniform temperature distribution, and precise control over the reaction parameters, facilitating the efficient and homogeneous doping of Mn and Al into the structure of the CQDs. This microwave-based strategy is expected to yield Al/Mn-doped CQDs with superior electrical properties compared to those synthesized via traditional methods^29^. Sugarcane bagasse (SB), a byproduct of sugarcane processing, poses a significant challenge for Egypt’s agricultural sector. It often accumulates in vast quantities, contributing to environmental issues such as pollution and land degradation. However, this seemingly problematic waste can be transformed into a valuable resource^30–32^. By leveraging this readily available agricultural byproduct, we can effectively transform SB into valuable carbon-based nanomaterials, such as CQDs^19^. Its accumulation leads to disposal issues, but its rich cellulose content offers a sustainable pathway for producing carboxymethyl cellulose (CMC).

In this work, SB is utilized as the precursor for CMC synthesis, a crucial step towards the subsequent fabrication of CQDs. The process involves alkaline treatment and etherification to convert the cellulose within SB into CMC, a water-soluble polymer with versatile applications. This CMC then serves as a carbon source for the synthesis of CQDs, providing a controlled and reproducible route for nanomaterial fabrication. By employing this approach, we not only address the environmental burden associated with SB waste but also demonstrate a sustainable, two-stage process: first, transforming SB into CMC, and then leveraging this CMC to produce high-value CQDs, which are subsequently doped with Mn and Al. This strategy underscores the potential of agricultural waste valorization, offering a circular economy approach to nanomaterial synthesis.

## Materials and methods

### Materials

The sugarcane bagasse (SB) was obtained from the Paper Industry Quena Company, Egypt. All chemicals used were of pure analytical-grade and were purchased from Sigma-Aldrich, USA, and used exactly as received.

### Preparation of carboxymethyl cellulose from sugarcane bagasse

SB was treated with 1.5% HCl and then with 20% NaOH for 2 h. Afterward, the lignin was removed by bleaching with HClO_2_. Finally, pure cellulose was mercerized in an isopropanol solvent for 3.5 h with 17.5% NaOH and monochloroacetic acid^13,17^.

### Aluminum/manganese/carbon quantum dot synthesis

This procedure is carried out in three stages:

#### Manganese/carbon quantum dot (Mn/CQDs) synthesis

Potassium permanganate (1.8 g) and carboxymethyl cellulose (CMC) (0.3 g) were combined in 100 mL of deionized water within a 250 mL Teflon-lined hydrothermal autoclave. Manganese acetate (0.9 g) was then added to the solution under continuous stirring until completely dissolved. The resulting homogeneous mixture was sealed in the autoclave and heated in an oven at a controlled temperature of 140 °C for 12 h. The heating and cooling rates were maintained at 5 °C/min. After the reaction, the autoclave was allowed to cool naturally to room temperature. The resulting product was washed repeatedly with deionized water and ethanol via centrifugation (i.e., at 8000 rpm for 10 min per cycle) to remove unreacted precursors and byproducts. Finally, the washed solid was dried in a vacuum oven at 60 °C for 24 h to obtain Mn/CQDs powder^26^.

#### Aluminum incorporation via microwave treatment (Al-Mn/CQDs 1)

The synthesized Mn/CQDs powder was dispersed in an aqueous solution of aluminum nitrate (AlNO₃) at a concentration of 100 ppm with a solid-to-liquid ratio of 1 mg/mL. This mixture was subjected to probe homogenization for 5 min to ensure uniform dispersion. Subsequently, the dispersed mixture was transferred to a microwave and irradiated in a domestic microwave oven at a power level of 70 W for 180 s (3 min). After microwave irradiation, the resulting black suspension was heated in an oven at 60 °C until complete evaporation of the solvent to obtain the Al-Mn/CQDs 1 dry powder.

#### Final drying (Al-Mn/CQDs 2)

The obtained Al-Mn/CQDs 1 dry powder was further dried in a muffle furnace at a controlled temperature of 105 °C for a duration of 6 h to ensure complete removal of any residual moisture and to obtain the final Al-Mn/CQDs 2 product. The heating and cooling rates of the muffle furnace were maintained at 10 °C/min.

### Characterization

Fluorescence microscopy was performed using a Jasco FP-6500 spectrofluorometer (made in Japan) with a 150-watt xenon arc lamp.

The UV–vis absorption spectrum was recorded by a UV–Vis spectrophotometer (JASCO V-630, Tokyo, Japan) using a 1 cm path length quartz cell. The quantum yield (QY) was calculated according to the formula:

1 $${\text{QY}} = {\text{Qst}}. \times \frac{{mx}}{{mst.}} \times \left( {\frac{{\upeta x}}{{\upeta st.}}} \right).$$

where “m” is the slope from the plot of fluorescence vs. absorbance, the “x” indicates the S, N–CQDs, and “st.” refers to methylene blue standard solution in water (0.1 M)^14,15^.

The band gap energy of the carbon dots was fundamentally determined by analyzing their absorbance and transmittance spectra. Both direct and indirect electronic transitions were evaluated using Tauc’s method Eq. (2), where A represents an optical constant, α is the absorption coefficient (cm⁻¹), hv signifies the photon energy, E_g_ is the band gap energy, and n is a transition-dependent exponent (n = 1/2 for direct transitions and n = 2 for indirect transitions). The absorption coefficient (α) was calculated according to Eq. (3), where ‘a’ denotes the absorbance and ‘d’ is the path length of the cuvette in centimeters^33^.

2 $${({\text{Ah}}\upsilon )^{{\text{1}}/{\text{n}}}}={\text{A}}({\text{A}}\upsilon-{{\text{E}}_{\text{g}}})$$

3 $$\:{\upalpha\:}\:=\:\frac{2.303\:\text{a}}{\text{d}}.$$

FTIR spectra were recorded using a Mattson 5000 spectrometer (Unicam, United Kingdom) with KBr pellets. The crystallinity index (LOI) and mean hydrogen bond strength (MHBS) were calculated using Eqs. (4) and (5).

4 $$\:\text{L}\text{O}\text{I}\:=\:\frac{{A}_{1425}}{{A}_{900}}$$

5 $$\:\text{M}\text{H}\text{B}\text{S}\:=\:\frac{{A}_{OH}}{{A}_{CH}}.$$

where A_1425_ and A_900_ refer to the FTIR absorbance at 1425 and 900 cm^− 1^, respectively. In addition, A_OH_ and A_CH_ refer to the FTIR absorbance of the OH and CH peaks, respectively^17,34^. Density functional theory (DFT) calculations were carried out using the Gaussian 09 W software package. Geometry optimization was performed using the Berny algorithm. Different parameters were investigated via DFT calculations, including some of the optimized geometries and ground state energies, including total energy (E_T_), the energy of the highest occupied molecular orbital (E_HOMO_), the energy of the lowest unoccupied molecular orbital (E_LUMO_), the energy gap (E_g_), the dipole moment (µ), the absolute hardness (η), the absolute softness (σ), the chemical softness (S), and the additional electronic charge (ΔN_max_)^14,17,35^.

6 $$\:{E}_{gap}=({E}_{LUMO}-{E}_{HOMO})$$

7 $$\:{\upeta\:}=\frac{({E}_{LUMO}+\:{E}_{HOMO})\:\:}{2}\:$$

8 $$\:{\upsigma\:}=\frac{1\:\:}{{\upeta\:}}$$

9 $$\:\text{S}=\frac{1\:\:}{2{\upeta\:}}$$

10 $$\:{\Delta\:}{N}_{max}=\frac{-\text{P}\text{i}\:\:}{{\upeta\:}}.$$

The samples’ dielectric properties were measured using an alpha impedance analyser, which is part of the broadband dielectric spectrometer, BDS, from NovoControl Co., Germany. BDS also contains a quatro-system for temperature control using N_2_ gas from a liquid N_2_ dewar. NovoControl provides the software Win Deta, which completely controls the measurement process.

## Results and discussion

### Optical, quantum, and mechanistic properties

The calculated QY values were 0.03, 1.02, and 42.65% for Mn/CQDs, Al-Mn/CQDs 1, and Al-Mn/CQDs 2, respectively, compared to 28.7% for N/Al-CDs^24^. This result highlights a significant enhancement in fluorescence efficiency upon aluminum incorporation followed by thermal treatment. While fluorescence in CQDs is often attributed to C = O moieties on their surfaces, and the shifts in emission peaks can be related to size variations, the substantial increase in QY for Al-Mn/CQDs 2 necessitates a more in-depth mechanistic explanation beyond these general observations. The initial increase in fluorescence intensity upon aluminum doping in Al-Mn/CQDs 1 compared to Mn/CQDs suggests that the introduction of Al³⁺ ions plays a crucial role in passivating non-radiative recombination pathways. These pathways, often associated with surface defects and dangling bonds, can quench fluorescence. The interaction of Al³⁺ with the CD surface, potentially through coordination with oxygen-containing functional groups, may reduce the density of these defect states, leading to a higher radiative decay rate and thus increased fluorescence intensity. The further dramatic enhancement in QY upon heating to 105 °C (Al-Mn/CQDs 2) likely involves a combination of structural and electronic modifications induced by the thermal treatment in the presence of aluminum.

**Fig. 1:**
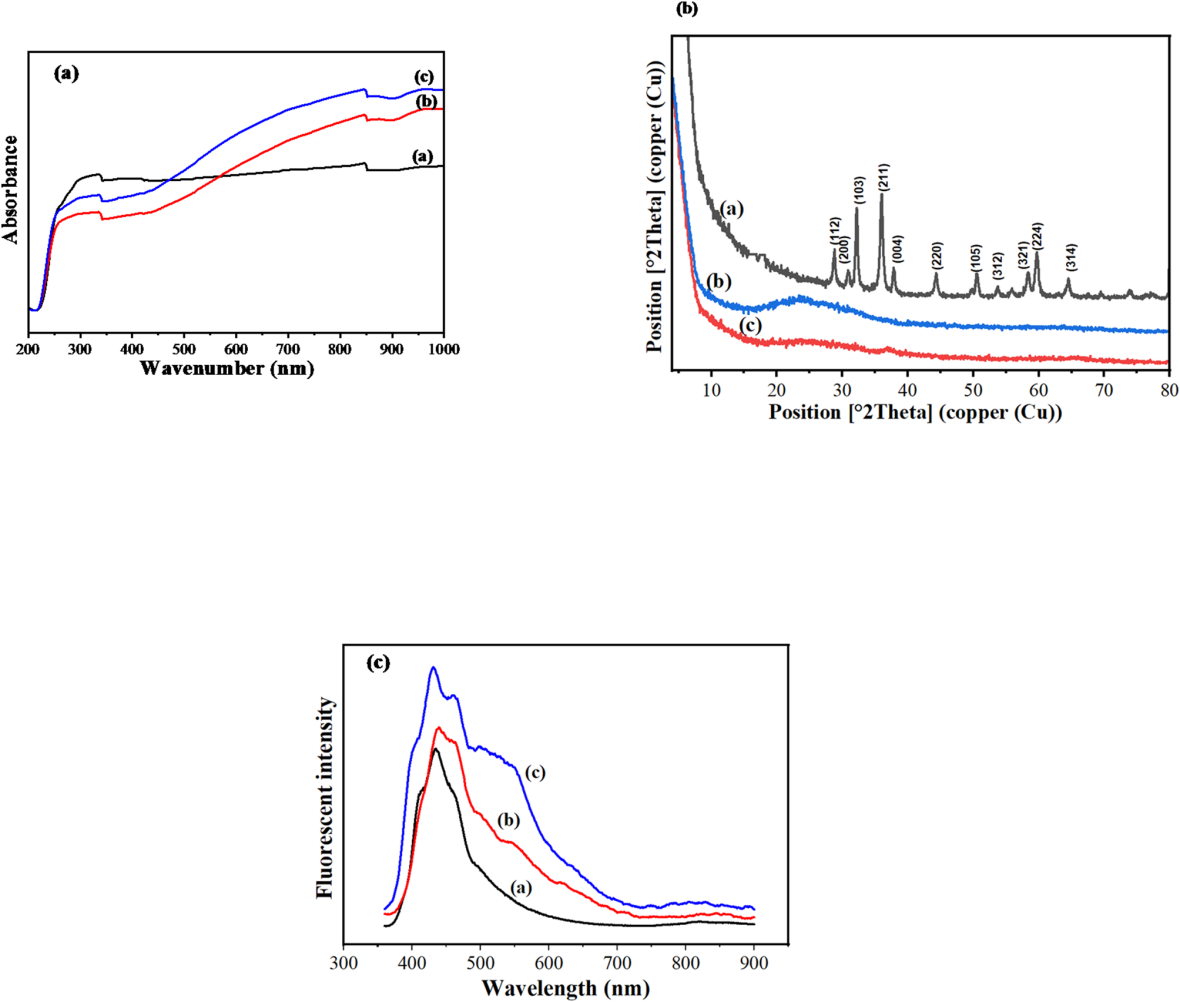
(**a**) UV spectra, (**b**) XRD spectra, and fluorescence spectra of; (**a**) Mn/CQDs, (**b**) Al-Mn/CQDs 1, and (**c**) Al-Mn/CQDs 2.

The UV absorbance spectra of Mn/CQDs, Al-Mn/CQDs 1 and Al-Mn/CQDs 2 in the range 200–1000 nm range are illustrated in Fig. 1a. As can be seen, a slight red shift is observed along with a significant enhancement in the absorbance in the visible region with Al doping in Mn/CQDs system. Crystal growth in a highly temperature atmosphere, combined with the presence of Al and carbon impurities, leads to a range of defect absorption bands observed in Al-Mn/CQDs 1 and Al-Mn/CQDs 2 ^36^.

The XRD analysis for Mn/CQDs yielded patterns consistent with the standard reference for Mn₃O₄ (JCPDS Card No. 00-001-1127), confirming the successful preparation of this manganese oxide phase^37^. The XRD of Al-Mn/CQDs 1 and Al-Mn/CQDs 2 showed a more amorphous structure. The more amorphous structure observed in the XRD patterns of Al-Mn/CQDs 1 and Al-Mn/CQDs 2 likely stems from the disruption of the carbon dot lattice by the introduction of Al³⁺ ions, which can hinder the formation of ordered graphitic domains by interacting with the surface and potentially promoting heterogeneous nucleation of smaller, less crystalline regions; furthermore, in Al-Mn/CQDs 1, the presence of aluminum nitrate precursors can mask any weak crystalline signals, while the subsequent low-temperature thermal treatment for Al-Mn/CQDs 2, primarily aimed at nitrate removal, is insufficient to induce significant carbon dot crystallization and may even enhance surface disorder, thus maintaining the overall amorphous nature characteristic of many carbon dot materials (Fig. 1b).

**Fig. 2:**
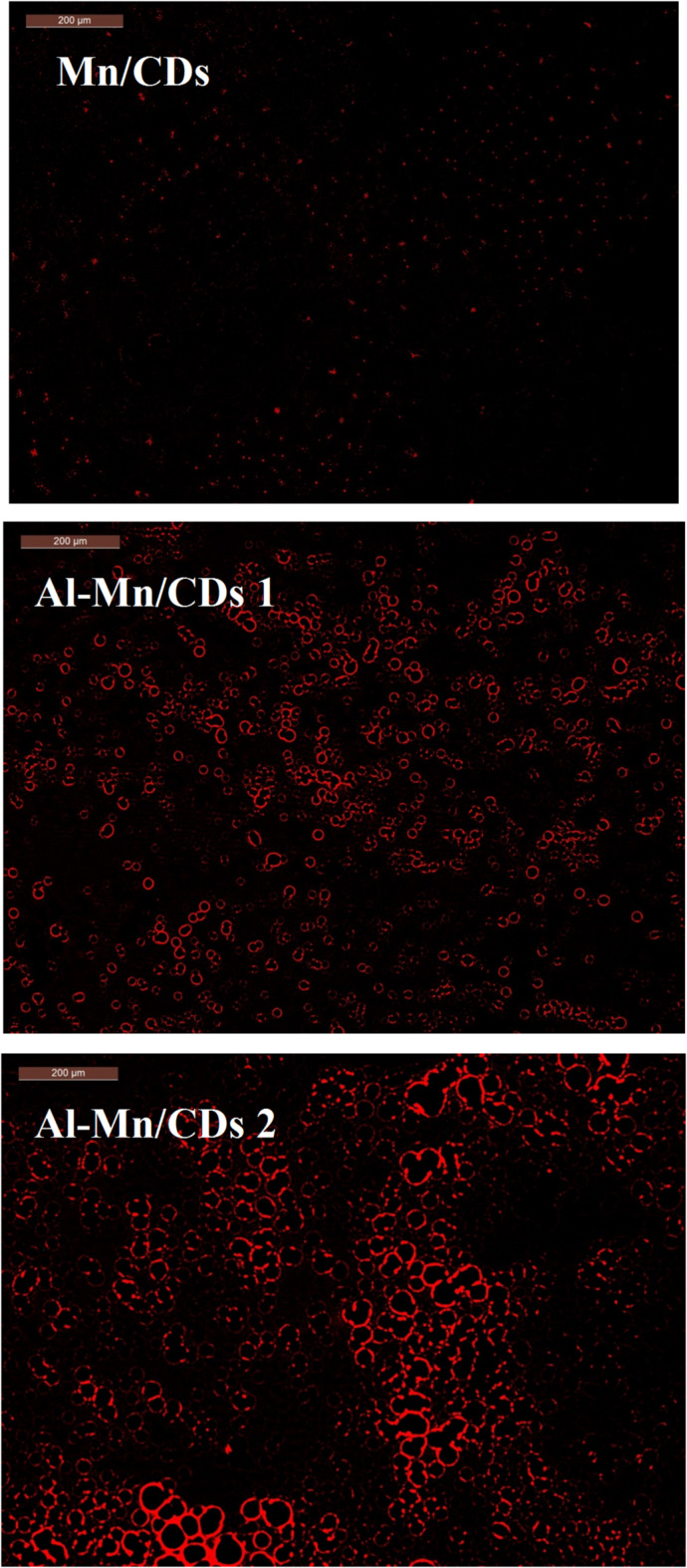
Fluorescence microscope of Mn/CQDs, Al-Mn/CQDs 1, and Al-Mn/CQDs 2.

The fluorescence spectroscopy was performed with an excitation wavelength of 350 nm and showed the maximum emission wavelengths of 438, 439, and 431 nm for Mn/CQDs, Al-Mn/CQDs 1, and Al-Mn/CQDs 2, respectively. Fluorescence emission is caused by the C = O moieties on the surfaces of CQDs^13,15^. The emission peaks at 496, 502, and 551 nm are derived from the oxygen vacancy defects in Mn/CQDs, Al-Mn/CQDs 1, and Al-Mn/CQDs 2, respectively. This difference in the position of the peaks is attributed to the variation in CQDs size^13,18^. The quality of the CQDs obtained is associated with the full width of half maximum (FWHM), calculated as 39, 45 and 87 nm, for Mn/CQDs, Al-Mn/CQDs 1 and Al-Mn/CQDs 2, respectively, which express the high quality of Al-Mn/CD 2 compared to Mn/CQDs, Al-Mn/CQD 1 and Al-Mn/CQD 2 (Fig. 1c). The fluorescence spectroscopy proved that the intensity of fluorescence increased after aluminum doping for Al-Mn/CQDs 1 compared to Mn/CQDs. At the same time, after increasing the temperature, the fluorescence intensity increased for Al-Mn/CQDs 2. Fluorescence microscope, Fig. 2, visually confirmed the enhanced emission in Al-Mn/CQDs 2, demonstrating a notably intense red fluorescence. This red emission was significantly stronger than that observed for Mn/CQDs and Al-Mn/CQDs 1, further supporting the observed trends in QY and FWHM.

The calculated band gap of Mn/CQDs, Al-Mn/CQDs 1, and Al-Mn/CQDs 2 by Tauc plot method equation was calculated to be 1.21, 0.7, and 0.3 eV, respectively. This indicates a decrease in the calculated energy gap as a function of the dopant types. The reduction in band gap upon aluminum doping in Al-Mn/CQDs 1 suggests that the introduction of Al atoms creates new energy levels within or near the band gap of the Mn/CQDs. This doping-induced modification can alter the electron-hole recombination pathways and potentially enhance the material’s interaction with lower energy photons, leading to changes in its optical absorption and emission properties. The further decrease in the band gap observed in Al-Mn/CQDs 2, which underwent an additional temperature treatment, indicates that thermal processing plays a crucial role in modifying the electronic structure of the aluminum-doped CQDs. The increased temperature might facilitate a stronger interaction or chemical bonding between the aluminum species and the carbon core, leading to the formation of new surface states or the alteration of existing ones. This finding has important implications for applications where specific emission wavelengths are required, such as in sensing, or LED development^38–40^.

### FTIR spectroscopy with DFT calculations

**Fig. 3:**
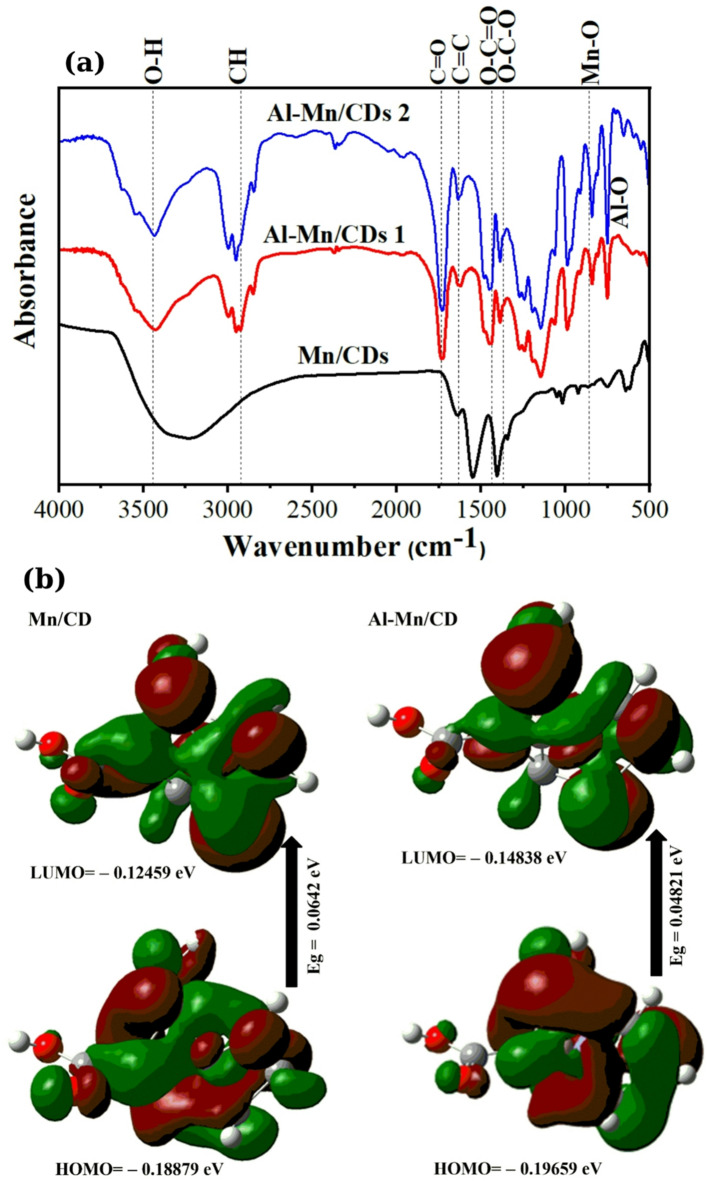
(**a**) FTIR spectra of Mn/CQDs, Al-Mn/CQDs 1 and Al-Mn/CQDs 2, and (**b**) DFT calculations of Mn/CQDs and Al-Mn/CQDs.

The FTIR spectra of the prepared Mn/CQD, Al-Mn/CQD 1 and Al-Mn/CQD 2 show absorption bands at 3270–3432 cm^− 1^ (O–H), 1641–1727 cm^− 1^ (C = O), 1546–1623 cm^− 1^ (C = C), 1405–1450 cm^− 1^ (C–O = C), 1342–1386 cm^− 1^ (C–O–C), and 746–844 cm^− 1^ (Mn–O) as depicted in Fig. 3a^26^. A new peak appeared between 750 and 752 cm^− 1^ related to the Al–O in the Al-Mn/CQD 1 and Al-Mn/CQD 2 ^41^.

The O–H groups exhibited a relative absorbance (RA) of 0.56, 1.01, and 1.02 for Mn/CQD, Al-Mn/CQD 1, and Al-Mn/CQD 2, respectively, which indicates that Al-Mn/CQD 2 is more hydrophilic than Mn/CQD and Al-Mn/CQD 1 ^14^. The calculated MHBS was 0.72, 1.01, and 1.10 for Mn/CQD, Al-Mn/CQD 1 and Al-Mn/CQD 2, respectively, which means a strong H-bond strength with the incorporation of aluminum and a further enhancement with increased synthesis temperature. The observed increase in MHBS upon aluminum incorporation into the CQDs can be attributed to the introduction of additional oxygen-containing functional groups on the CQD surface. Aluminum ions (Al³⁺) possess a high affinity for oxygen, facilitating the formation of hydroxyl (-OH) and carboxyl (-COOH) groups^42,43^. These oxygen-rich moieties act as potent H-bond donors and acceptors^44^significantly enhancing the interaction between the CQDs and water molecules. Consequently, the number of potential H-bonding sites increases, resulting in a higher MHBS value for Al-Mn/CQDs 1 compared to Mn/CQDs. While both Al-Mn/CQDs 1 and Al-Mn/CQDs 2 contain aluminum, the latter underwent synthesis at a considerably higher temperature. This elevated temperature likely facilitated a more extensive and efficient incorporation of aluminum into the CD structure, leading to a greater density of oxygen-containing functional groups, such as hydroxyl and carboxyl groups, on the CD surface. These functional groups are pivotal for H-bond formation with water molecules.

To further elucidate the regulation of the electronic band structure upon aluminum incorporation, we delve into the orbital interactions revealed by our DFT calculations, Fig. 3b. The observed changes in the HOMO and LUMO energy levels, and consequently the energy gap (Eg), are a direct consequence of the hybridization between the atomic orbitals of aluminum and the constituent atoms (carbon, manganese and aluminum) of the carbon dots (Fig. 3b; Table 1). Firstly, the dipole moment (µ) of Al-Mn/CQDs (2.50 Debye) was substantially lower than that of Mn/CQDs (2.54 Debye), as determined through DFT calculations, which arises from fundamental alterations in the electronic structure and molecular geometry upon the introduction of aluminum. DFT provides a detailed map of electron density, revealing that the presence of Al significantly reshapes the charge distribution within the carbon dots. Aluminum, with its distinct electronegativity compared to manganese and carbon, influences how electrons are shared amongst the atoms. This variation in electron sharing leads to a redistribution of charge, potentially resulting in a more balanced or less polarized arrangement, thereby lowering the overall dipole moment. Secondly, the calculated energy gap (E_g_) for Al-Mn/CQDs (0.0482 eV) is lower than that of Mn/CQDs (0.0642 eV). This reduction in Eg indicates that the Al-Mn/CQDs material requires less energy to excite electrons from the valence band to the conduction band, effectively enhancing its conductivity. The introduction of aluminum, with its distinct electronic configuration, creates new electronic states within the band structure. These states can modify the positions and shapes of the valence and conduction bands, resulting in a narrowed energy gap. Furthermore, the presence of aluminum can generate defect states or impurity levels within the band gap, acting as intermediate energy levels that facilitate electron transitions, thereby reducing the overall energy required for excitation^45^. The lowering of the LUMO energy level in Al-Mn/CQDs (– 0.14838 eV vs. – 0.12459 eV for Mn/CQDs) suggests that the introduction of aluminum creates new acceptor states or modifies existing ones to be more energetically favorable for accepting electrons. This can occur through the interaction of the relatively low-lying empty p-orbitals of Al³⁺ with the π* antibonding orbitals of the carbon framework. This interaction stabilizes the LUMO, effectively lowering its energy. Conversely, the slight lowering of the HOMO energy level in Al-Mn/CQDs (– 0.19659 eV vs. – 0.18879 eV for Mn/CQDs) indicates a stabilization of the highest occupied molecular orbitals. This stabilization can arise from the inductive effects of the aluminum ion, which, due to its different electronegativity, can redistribute electron density and influence the energy of the π bonding orbitals. Additionally, potential bonding interactions between aluminum and oxygen-containing functional groups on the CQD surface can also contribute to this stabilization. The consequent reduction in the E_g_ is a direct outcome of these shifts in the HOMO and LUMO levels^46^. Moreover, aluminum doping introduces new electronic states and modifies the existing ones through orbital mixing, effectively narrowing the energy difference between the highest occupied and lowest unoccupied levels. This narrowing facilitates electronic transitions and explains the enhanced conductivity observed in Al-Mn/CQDs. Thirdly, the significantly higher softness of Al-Mn/CQDs (20.74 eV) compared to Mn/CQDs (15.57 eV) indicates a substantial change in the polarizability and reactivity of the CQDs upon the introduction of aluminum. Softness, in this context, is a measure of how easily the electron cloud of a molecule can be distorted or polarized. A higher softness value implies that the Al-Mn/CQDs are more susceptible to electronic perturbations, making them more reactive and capable of forming stronger interactions with other species. Aluminum doping modifies the electron distribution in Al-Mn/CQDs, creating a more diffuse and easily polarized electron cloud with new electronic states near the Fermi level. This increased softness and polarizability facilitate stronger charge transfer, enhancing charge injection, extraction, conductivity, and sensor sensitivity. Consequently, these improved electrical properties make Al-Mn/CQDs highly suitable for catalytic, electrochemical, and other advanced electronic applications.

**Table 1:** The quantum chemical parameters of mn/cd and Al-Mn/CD.

DFT B3LYP/6–31G (d)	Mn/CD	Al-Mn/CD
E_LUMO_ (eV)	− 0.12459	− 0.14838
E_HOMO_ (eV)	− 0.18879	− 0.19659
E_g_ (eV)	0.0642	0.0482
E_T_ (au)	− 1638.16	− 1874.34
µ (Debye)	2.54	2.50
ɳ (eV)	0.0321	0.0241
σ (eV)	31.15	41.48
S (eV)	15.57	20.74

The properties observed in Al-Mn/CQDs, specifically the significantly lower E_g_ and substantially higher softness compared to Mn/CQDs, strongly suggest an enhancement of their electrical properties, making them promising candidates for various electronic applications. The reduced energy gap is a critical factor in improving conductivity, as it signifies a lower energy barrier for electron excitation from the valence band to the conduction band. This facilitates easier electron movement within the material, translating to higher electrical conductivity. Consequently, Al-Mn/CQDs are better suited for applications requiring efficient charge transport, such as in electronic devices, solar cells, and sensors, where minimizing energy loss during charge transfer is paramount. This stabilization often results in enhanced charge transfer and conductivity because the lowered energy state allows electrons to be more easily excited or transferred. Essentially, the more negative E_T_ reflects an energetically favorable configuration, which can lead to improved performance in electronic and catalytic applications. Finally, the lower total energy (E_T_) measured in atomic units (au) of Al-Mn/CQDs (–1874.34 au) compared to Mn/CQDs (–1638.16 au) provides crucial insights into the stability and formation of the aluminum-modified carbon dots. In thermodynamics, a lower E_T_ signifies a more stable system, indicating that the Al-Mn/CQDs structure is energetically more favorable than its unmodified counterpart. This suggests that the incorporation of aluminum into the Mn/CQDs framework results in a more stable arrangement of atoms and electrons. Furthermore, the reduced E_T_ often implies the presence of stronger bonding interactions within the Al-Mn/CQDs structure. This could be attributed to the formation of stronger Al-C or Al-Mn bonds, which contribute to the overall stability of the system^47^. This energetic favorability also aligns with the observed sheet-like structures in TEM, which likely represent a more stable configuration facilitated by aluminum. Finally, the energy difference between the two systems suggests that the formation of Al-Mn/CQDs from Mn/CQDs and aluminum is an exothermic process, releasing energy and indicating that the reaction is likely to proceed spontaneously^48^. In summary, the lower total energy of Al-Mn/CQDs signifies a more stable and energetically favorable structure, likely due to stronger bonding interactions and a favorable formation process, highlighting the beneficial impact of aluminum incorporation.

### Morphological analysis

**Fig. 4:**
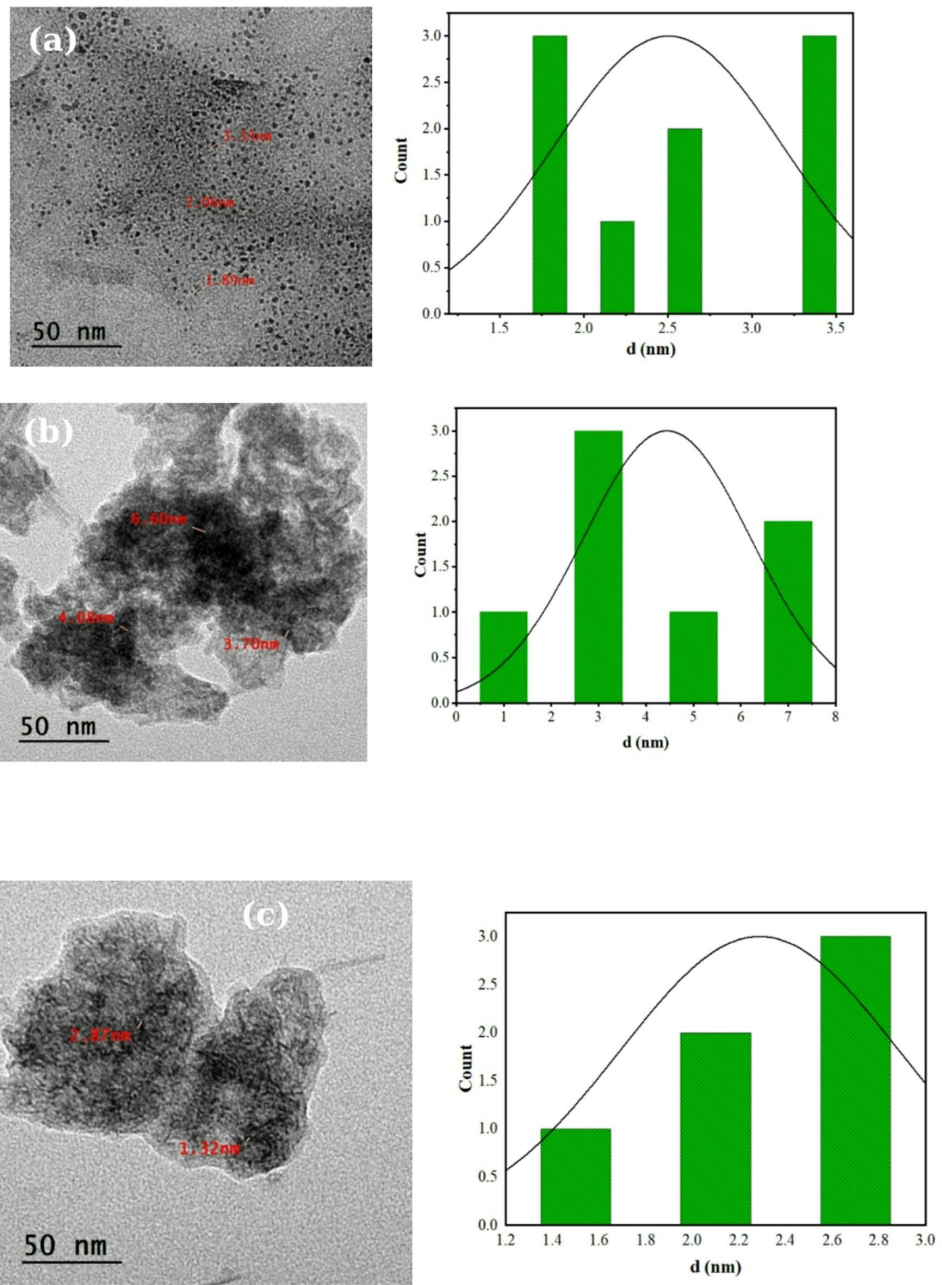
Analysis of (**a**) Mn/CQDs, (**b**) Al-Mn/CQDs 1, and (**c**) Al-Mn/CQDs 2 with pore size distribution.

TEM analysis of Mn/CQD, Al-Mn/CQD 1, and Al-Mn/CQD 2 revealed that the CQDs were spherical as shown in Fig. 4a-c. The particle size range was 1.89–3.33 nm for Mn/CQD, 3.70–6.60 nm for Al-Mn/CQD 1, and 1.32–2.87 nm for Al-Mn/CQD 2. The observed increase in particle size for Al-Mn/CQD 1 can be attributed to heterogeneous nucleation and growth facilitated by the presence of Al³⁺ ions on the surface of the Mn/CQDs. These ions can act as nucleation sites, promoting the deposition of more carbonaceous material and potentially leading to inter-particle interactions and aggregation during the synthesis process, resulting in larger average particle sizes.

The subsequent decrease in particle size for Al-Mn/CQD 2 after thermal treatment at elevated temperatures is likely due to the decomposition and volatilization of aluminum nitrate precursors and loosely bound surface species. This process can lead to a reduction in the overall mass and size of the carbon dot structures. Furthermore, the thermal energy can induce surface restructuring and fragmentation of larger, less stable aggregates formed during the aluminum incorporation stage, resulting in a population of smaller, more uniform nanoparticles. The removal of surface nitrates can also reduce electrostatic repulsion between particles, potentially allowing for some degree of size refinement driven by Ostwald ripening, where smaller particles dissolve and redeposit onto larger ones, although the overall trend here is a decrease in size, suggesting a more controlled aggregation process.

### Dielectric properties of the samples

**Fig. 5:**
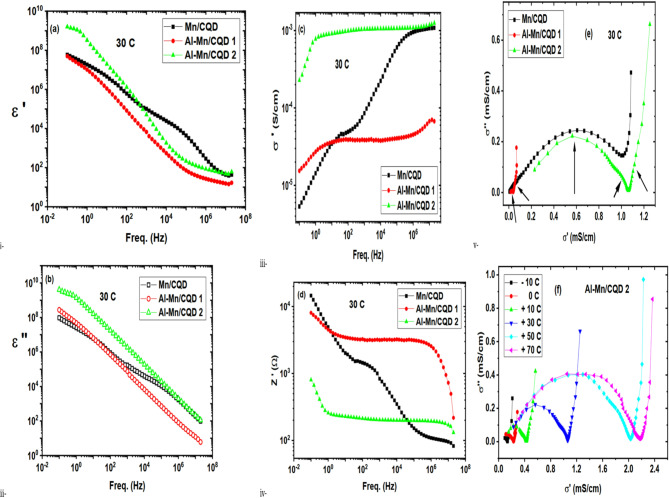
(**a**) Permittivity versus frequency, (**b**) Loss versus frequency, (**c**) Conductivity versus frequency, (**d**) Real impedance versus frequency, (**e**) Cole–Cole plot of conductivity for Mn/C QDs, Al-Mn/CQDs 1, and Al-Mn/CQDs 2, and (**f**) Cole–Cole plot of conductivity for Al-Mn/CQDs 2 at different temperatures.

In Fig. 5a, the permittivity illustrates that adding Al nitrates and lowering the drying temperature decrease the permittivity. Decreasing the drying temperature did not allow the nitrates to evaporate, which leaves the nitrate groups attached chemically and physically to the surrounding molecules. In other words, this weakens the ability of the composite to store charges. Drying the sample at a higher temperature increased the permittivity by about one order of magnitude at a lower frequency range (up to the ionic movement frequency, 100 Hz). This tells us that the higher drying temperature provided enough energy to the nitrate groups to evaporate. The departure of nitrate groups resulted in numerous vacancies in the surrounding environment and left Al atoms free. These effects led to increased ionic movement and charge storage within the sample at a lower frequency range. In Fig. 5b, the loss or the imaginary part of the permittivity represents the flow of charge within the sample. It is clear that Al nitrates, especially in samples dried at a higher temperature, increased charge transfer levels significantly for a wide frequency range. Data also reveal that drying the sample at 60 °C was not enough to liberate the nitrate groups. Consequently, they interacted with the sample structure, tying more knots among the atoms and molecules. This causes a decrease in permittivity and loss, as well as a reduction in free charge transfer^49^. Conductivity data, Fig. 5c, indicate that Al nitrates resulted in a stable conductivity level, except for the electrode polarization at low frequency. The presence of nitrate groups entangles molecules and limits free charge movement^49,50^.

Embedding Al nitrate within the composite led to a stable impedance across a wide frequency range, Fig. 5d. Heating the sample during the addition of aluminum nitrate led to the removal of nitrate groups, thereby freeing the aluminum ions to move and interact without constraint. This causes a significant reduction in the impedance and a better preservation of the impedance stability across the attained frequency range. Adding Al nitrates and dehydrating the Mn/CQDs sample at 60 °C introduces another conduction mechanism as nitrate groups form chemical and/ or physical bonds with other composite components. Moreover, dehydrating the composite at 105 °C after the addition of Al nitrates caused the removal of at a considerable amount of the nitrate as a vapor, leaving Al atoms free to combine with the composite through bonding and/ or move freely, acting as free charge carriers. In Fig. 5e, the Cole-Cole conductivity plot indicates that the Mn/CQDs composite has only one conduction mechanism, as it forms only one semicircle. Whereas the addition of Al to the composite in a small ratio, as indicated in the material and methods, results in two semi-circles, referring to the introduction of a new conduction mechanism that involves Al, as indicated by the two arrows. Further addition of Al led to the initiation of a third conduction mechanism at higher frequencies. These data indicate that Al is responsible for the two conduction mechanisms occurring at higher frequencies. The increase in conduction mechanisms, through **an increase in the** types of charge carriers, explains the reduced impedance and the stable impedance across the wide frequency range. Nyquist plot of conductivity for Al-Mn/CQDs 2 indicates two interfering conduction mechanisms, both of which enhance the sample’s conductivity and exhibit semiconductor behavior (Fig. 5f). In other words, the conductivity is proportional to temperature. As conductivity increased from less than 0.2 mS/cm at − 10 °C to 1.05 and 2.2 mS/cm at 30 °C and 70 °C, respectively^50^.

Electrical data of the prepared samples prove the significance of the different additions used in the study. Authors could improve the conductivity to reach a stable level over seven decades of frequency and achieve a low, stable impedance. Increasing permittivity and conductivity enhances the composite’s ability to store electric charges. Consequently, more dipoles are formed, allowing for more electrons to be available in the medium to eliminate static charges and interact with electromagnetic waves through multiple reflections and eventually absorption by the dipoles provided by the increased permittivity^51–58^. The prepared composite, derived from recycled sugar cane cellulosic by-products, exhibits high permittivity, superior conductivity, and stability across a wide frequency range. These properties make it ideal for eco-friendly applications. For instance, its anti-static behavior ensures clean room floors remain free of static discharge and protects electronic packaging and sensitive components during manufacturing^59^. Moreover, its stable impedance qualifies the composite as a standard for calibrating RLC bridges, while its robust electrical performance supports load matching, current limiting, resistive functions, and electromagnetic interference shielding through multiple reflections^60^.

## Conclusion

In this study, aluminum/manganese-doped carbon quantum dots (Al-Mn/CQDs) were successfully synthesized from sugarcane bagasse, a readily available agricultural waste, thus offering a sustainable and eco-friendly approach to nanomaterial production. The synthesis process, employing a microwave-assisted method, yielded Al-Mn/CQDs with enhanced electrical and optical properties, demonstrating their potential for diverse applications. Optical characterization revealed a significant enhancement in fluorescence intensity and quantum yield (QY) in Al-Mn/CQDs, particularly in the sample subjected to a higher temperature treatment (Al-Mn/CQDs 2). This sample exhibited a QY of 42.65%, a substantial improvement over Mn/CQDs and Al-Mn/CQDs 1. The strong red fluorescence emission observed in Al-Mn/CQDs 2, along with its narrow full width at half maximum (FWHM), highlights its potential for applications in bioimaging, sensing, and optoelectronic devices. Furthermore, the introduction of aluminum significantly altered the structural and electronic properties of the CQDs. TEM analysis showed a transition from well-dispersed spherical CQDs in Mn/CQDs to aggregated, sheet-like structures in Al-Mn/CQDs, indicating a change in self-assembly behavior. DFT calculations corroborated these findings, revealing a lower energy gap (E_g_) and higher softness in Al-Mn/CQDs compared to Mn/CQDs. These properties suggest enhanced electrical conductivity and charge transfer capabilities, making Al-Mn/CQDs promising for electronic applications. Dielectric measurements confirmed the improved electrical properties of Al-Mn/CQDs, showing stable conductivity and low impedance across a wide frequency range. In summary, this study highlights the versatility of Al-Mn/CQDs derived from sugarcane bagasse, showcasing their potential in both electrical and optical applications. The combination of enhanced electrical conductivity, stable impedance, and strong fluorescence emission makes these materials highly promising for future technological advancements, contributing to a more sustainable and technologically advanced future.

## Data Availability

Data is provided within the manuscript.
